# Two Cases of Death, Apparently from Eating a Great Number of Eggs

**Published:** 1817-07

**Authors:** R. Morrison

**Affiliations:** R. N.


### *Two Cases of Death, apparently from eating a great Number of Eggs.* By R. MORRISON, Esq. R. N.

ON the 26th of December, 1815, Charles Atherton and Henry Chalmers, mariners, were entered on the sick list, complaining of having been much troubled in their bowels during the preceding night; with alternate heats and chills; pain in the head, back, and joints; pain and tension in the epigastric region; with other febrile symptoms. They both attributed their complaints to cold, though it afterwards was proved, that on the preceding day they had eaten *forty-four boiled eggs* between them!

CASE 1. I shall offer a sketch of their symptoms separately. To Charles Atherton I gave two drachms of the sulphate of magnesia, every two hours, till three loose and naturally-looking stools were procured. The febrile symptoms, instead of being mitigated by this, appeared to be increased. He was bled from the arm to sixteen ounces, which was all that he could bear. It exhibited no buff, nor other unhealthy appearance. A dose of calomel and antimonial powder was given him at bed-time.

27th. Has passed a restless night. Had a partial diaphoresis confined to the face, neck, and arms. His countenance is dejected, and he gives himself up, in a very unusual degree, to despondency. Pulse weak and quick; tongue dry and furred; pain in the umbilical region. Sumat statim ol. ricini ℥j.—5 P. M. The oil operated twice. Still complains of great pain in the epigastrium and heat. Now related the circumstance of the egg feast for the first time. There is this evening a considerable exacerbation of fever, and another venæsection was employed. On the abstraction of sixteen ounces of blood deliquium animi took place.

28th. Has had hiccup and vomiting all night, which could not be restrained by effervescing draughts, opium, &c. and he expired this morning, not quite sixty hours from the time of his eating the eggs. From circumstances, unnecessary to mention, dissection could not be obtained.

CASE 2. Henry Chalmers (26th of December) complained of precisely the same symptoms as his companion, and had the sulphate of magnesia as in the former instance.

27th. The salts operated freely, and upon the whole he has passed a tolerable night. Complains this morning of a universal sense of extreme debility. His countenance is much dejected, as in the other patient, and despondency prevails. The vascular action is so depressed, that I dare not venture on venæsection. Habeat subm. hyd. et pulv. antim āā gr. iv. h. s. s.

28th. Passed a bad night. Has been sick at stomach several times, and vomited a little. *Eyes and face tinged quite yellow this morning.* Complains of great pain in the epigastrium, and keeps drawing up his legs towards the abdomen. Pulse very small and quick; thirst urgent. To take a scruple of calomel and two grains of opium immediately.

29th. The vomiting returned in the night, and gastric irritability increases. This morning passed by stool three dead teres lumbricoides, each about four inches in length. The calomel and opium to be repeated. 5 P. M. No stool since morning. Pain in the epigastric region less severe. Habeat statim ol. ricini ℥j.

30th. Passed a better night, and had one inconsiderable motion from the castor oil. To take two grains of calomel every six or eight hours.

January 2d, 1816. Since last report, he has continued the calomel in small doses, at greater or shorter intervals; and his mouth is now affected. But emaciation has made rapid strides; his eyes are sunk; his countenance cadaverous; hiccups, &c. Died at 10 o'clock this evening, eight days after the egg feast.

*Sectio Cadaveris.* The omentum was of a dark red colour, shrivelled and inflamed. The stomach was distended, partly with air, and partly with a dark dirty-looking fluid, of the most horrible and intolerable fœtor. The inner coat of the stomach was corrugated, and its vessels partially filled with blood. No decisive mark of inflammation in this viscus. The liver enormously gorged, and enlarged, apparently, to double its natural size. In texture soft, flabby, and seemingly rotten. The gall-bladder filled with dirty green-coloured bile. On the surface of the intestines, which were quite empty, were several dark-coloured spots; but no marks of adhesive inflammation between the different convolutions. In the thorax, no mark of recent disease, there being only old adhesions in the right cavity. Liquor pericardii small in quantity and pale. No morbid symptom in the head or other part of the body.

REMARKS.

That these patients both died from the same cause there can be little doubt; and although the first case terminated with much greater rapidity than the second, dissection would, in all probability, have developed a similar state of the abdominal viscera. It is somewhat singular that the liver should have borne the onus of the disease. The great pain in the epigastric region, in both cases, pointed out this viscus and the stomach as suffering from the effects of the imprudent act which these unhappy men had committed. I trust that the above Cases are worthy of record, as the practice of making enormous meals of eggs, at particular times in the year, is not uncommon in this country; and sailors are very apt to indulge in this luxury on coming in from sea. In hot climates eggs seem to have a very bad effect on the hepatic system in particular.

London, Feb. 27th, 1817.

